# Mechanism of Malondialdehyde-Induced Deterioration in Water-Holding Capacity of Bovine Myofibrillar Proteins: Insights from Structural Modifications and Molecular Docking

**DOI:** 10.3390/foods15173022

**Published:** 2026-08-27

**Authors:** He Li, Zhenyu Fang, Qin Wu, Dunhua Liu, Jun Liu, Ningxia Bu

**Affiliations:** 1 College of Life Sciences, Hubei Normal University, Huangshi 435002, China; lih10240217@163.com (H.L.);; 2 College of Food Science and Engineering, Hainan University, Haikou 570228, China; 3 College of Fisheries, Huazhong Agricultural University, Wuhan 430070, China; 4 College of Food Science and Engineering, Ningxia University, Yinchuan 750021, China; 5 Ningxia Veterinary Drugs and Fodder Inspection Institute, Yinchuan 750011, China

**Keywords:** oxidation modification, protein structure, lipid oxidation products, cysteine, water mobility

## Abstract

Lipid peroxidation products can induce oxidative modification of myofibrillar proteins (MPs), thereby compromising their water-holding capacity (WHC), but the underlying molecular mechanism remains unclear. This study systematically investigated the mechanism of MDA-induced WHC deterioration in bovine myofibrillar proteins (MPs) using multispectral techniques, redox proteomics, and molecular docking. Results demonstrated that low MDA concentrations caused relatively limited water release. Structural alterations became evident at 2 mM, whereas pronounced WHC deterioration occurred at 5–10 mM. At these higher concentrations, centrifugal loss increased by up to 48.80%, and immobilized water migrated to free water (*p* < 0.05). This functional decline was accompanied by substantial structural remodeling, characterized by a transition from α-helix to β-sheet conformations, decreased hydrogen bonding, and enhanced disulfide-associated cross-linking. Furthermore, redox proteomics identified 581 differential cysteine redox sites, including 343 increased and 238 decreased sites. Among these sites, 57 markedly decreased sites associated with myofibrillar and cytoskeletal proteins were further characterized, including sites in actin, α-actinin, myosin, and LIM-domain-containing proteins. Motif analysis further revealed a characteristic cysteine-rich C-x-x-C-x-C sequence pattern surrounding responsive oxidation sites. Molecular docking of 12 representative cysteine sites supported the spatial feasibility of MDA pre-association near these cysteine-containing regions, with actin C258 exhibiting the most negative docking score among the examined sites (−3.3 kcal/mol). These findings reveal that cysteine redox remodeling was associated with structural reorganization and increased water mobility, thereby contributing to WHC deterioration.

## 1. Introduction

Water-holding capacity (WHC) is a critical determinant of the eating quality, processing properties, and economic value of fresh meat and meat products [1]. Approximately 85% of the water in muscle is physically entrapped within the three-dimensional network formed by myofibrillar proteins (MPs). Therefore, the spatial conformation of MPs, the balance of intermolecular forces, and the stability of their network structure directly govern the water retention capacity of meat [2]. However, during slaughtering, processing, and storage, lipid peroxidation is almost inevitable, and its primary and secondary reactive oxidation products exert profound adverse effects on the structure and functionality of MPs [3]. Reactive carbonyl compounds include reactive aldehydes and ketones generated from lipid degradation. Because of their pronounced electrophilicity, these compounds readily react with key nucleophilic sites in proteins and thereby promote oxidative damage and changes in protein structure and function [4].

Among the amino acid residues targeted during lipid-protein co-oxidation, cysteine is particularly important because its thiol group is both highly nucleophilic and redox-sensitive [5]. Reactive oxygen species generated during lipid oxidation can convert cysteinyl thiols into sulfenic intermediates and disulfide bonds. Under severe oxidative conditions, they can also produce less reversible higher oxidation states. Electrophilic lipid-derived aldehydes may additionally interact with or modify accessible nucleophilic residues, including thiol-containing sites. Consequently, disruption of thiol-disulfide homeostasis can alter intramolecular and intermolecular cross-linking, promote protein unfolding or aggregation, and modify the surface charge, hydrophobicity, solubility, and filament organization of MPs. In muscle proteins, excessive loss of free sulfhydryl groups and site-specific cysteine oxidation have been associated with destabilization of secondary and tertiary structures. The resulting contraction and disorganization of the myofibrillar matrix reduce the accessibility of hydrophilic water-binding sites and weaken the spatial constraints on water. These changes facilitate water migration and exudation [5,6,7]. Thus, cysteine residues may constitute molecular redox hotspots that connect lipid-derived oxidative stress with structural deterioration and the loss of WHC in meat.

Malondialdehyde (MDA), one of the major secondary products derived from the peroxidation of polyunsaturated fatty acids, contains two highly reactive aldehyde groups and is therefore frequently regarded as a potent cross-linking agent capable of undergoing nucleophilic addition or Schiff base reactions with nucleophilic amino acid side chains in proteins, such as the sulfhydryl groups of cysteine and the ε-amino groups of lysine [8]. Moderate oxidation may slightly enhance gel strength in some systems through the formation of disulfide bonds. Excessive MDA accumulation, however, generally induces extensive cross-linking and aggregation of MPs, abnormal exposure of internal hydrophobic groups, and a marked reduction in protein solubility [9]. Such irreversible structural remodeling severely disrupts the capillary network responsible for water entrapment, promotes the migration of bound water and immobilized water toward free water, and ultimately results in substantial deterioration of WHC [1].

Although studies of lipid–protein co-oxidation systems have been reported, the molecular mechanisms underlying the MDA-mediated decline in MPs’ water-holding capacity, particularly the cysteine redox sites that respond to MDA and their relationships with protein structural remodeling, remain insufficiently understood. Accordingly, this study aimed to determine how MDA affects the WHC of bovine MPs and to assess whether cysteine redox remodeling is associated with structural and functional deterioration.

## 2. Materials and Methods

### 2.1. Extraction of Myofibrillar Proteins (MPs)

Quadriceps femoris muscles were obtained from six Huangpi yellow cattle (24–30 months of age; 500 ± 20 kg) purchased from Hubei Niuxiaomei Agricultural Technology Co., Ltd. (Huangshi, China). The cattle were slaughtered by the commercial supplier under its routine commercial procedure. Muscle samples were packed in polyethylene zip-lock bags, transported to the laboratory on ice, and immediately used for MPs extraction. MPs were extracted according to Li et al. [6] and Liu et al. [7], with slight modifications. Visible fascia and connective tissue were removed, and 50.00 g of muscle was homogenized for 90 s in an ice-water bath with 200 mL of pre-chilled extraction buffer (0.1 mol/L NaCl, 10 mmol/L NaH_2_PO_4_, 2 mmol/L MgCl_2_, and 1 mmol/L EGTA; pH 7.0) at a tissue-to-buffer ratio of 1:4 (*w*/*v*). The homogenate was centrifuged at 2000× *g* for 15 min at 4 °C, and the supernatant was discarded. The precipitate was resuspended in fresh extraction buffer, homogenized, and washed by centrifugation three times under the same conditions. The final suspension was filtered through four layers of defatted gauze (Huaxi Sanitary Material Co., Ltd., Xinxiang, China) and adjusted to pH 6.0 with 0.1 mol/L HCl. The resulting MPs precipitate was then collected.

### 2.2. Preparation of MDA

MDA was freshly prepared by acid hydrolysis of 1,1,3,3-tetramethoxypropane (TMP). A mixture of 8.4 mL of TMP, 10.0 mL of 5.0 mol/L HCl, and 31.6 mL of 50 mmol/L MES buffer (pH 6.0) was placed in a glass-stoppered bottle and incubated at 40 °C for 30 min in the dark. After cooling to room temperature, the hydrolysate was adjusted to pH 6.0 with 6.0 mol/L NaOH and brought to a final volume of 250 mL with 50 mmol/L MES buffer (pH 6.0). An aliquot was appropriately diluted, and its absorbance at 267 nm was measured using a UV-visible spectrophotometer (UV-1900i Plus, Shimadzu Corporation, Kyoto, Japan). MDA concentration was calculated using a molar extinction coefficient (ε_267_) of 31,500 M^−1^ cm^−1^ [10,11].

### 2.3. Preparation of the MDA-Treated MPs System

The MDA-treated MPs system was prepared according to previously reported methods with slight modifications [7,10,11]. Freshly extracted MPs were dispersed in PBS (pH 7.4) and adjusted to a protein concentration of 20 mg/mL. Freshly prepared MDA solution was then added to obtain final MDA concentrations of 0, 0.5, 1, 2, 5, and 10 mM. The mixtures were immediately transferred to tightly sealed glass vials and incubated at 25 °C for 24 h in the dark. Following incubation, the samples were washed with PBS by repeated resuspension and centrifugation at 2000× *g* for 10 min at 4 °C for three cycles, with the supernatant discarded after each cycle to remove unbound MDA. The resulting MPs fractions were collected, readjusted to the required protein concentration when necessary, and used for subsequent analyses.

### 2.4. Determination of Moisture Content and Centrifugal Loss

For moisture determination, 5.00 g of MPs sample (*m*_1_) was placed in a pre-weighed weighing bottle (*m*_0_). Samples were frozen at −80 °C for 30 min and subsequently freeze-dried under vacuum for 48 h to constant mass. The combined mass of the bottle and dried sample was recorded as *m*_2_. Moisture content was calculated as follows:

Moisture content (%) = [*m*_1_ − (*m*_2_ − *m*_0_)]/*m*_1_ × 100.

For centrifugal loss, 2.00 g of MPs sample was weighed before centrifugation (*m*_0_) and centrifuged at 1000× *g* for 10 min at 4 °C. The expressed water was carefully removed, and the remaining sample was reweighed (*m*_1_).

Centrifugal loss (%) = (*m*_0_ − *m*_1_)/*m*_0_ × 100.

### 2.5. Low-Field Nuclear Magnetic Resonance (LF-NMR) Analysis of Water Distribution

Water distribution and migration within the samples were characterized according to a previously reported protocol [7] using a LF-NMR analyzer (NMI20-015V-I, Niumag Analytical Instrument Co., Ltd., Suzhou, China). MPs samples were placed into NMR tubes, and the probe temperature was maintained at 32 °C. The transverse relaxation time (T_2_) was measured using a Carr Purcell Meiboom Gill (CPMG) pulse sequence. Based on the inversion-reconstructed T_2_ spectra, the relative proportions and distribution changes in bound water (T_21_), immobilized water (T_22_), and free water (T_23_) were determined and analyzed.

### 2.6. Sodium Dodecyl Sulfate–Polyacrylamide Gel Electrophoresis (SDS-PAGE)

The concentration of MPs samples was adjusted to 5 mg/mL, after which the samples were mixed with four volumes of loading buffer (4% SDS, 20% glycerol, 10% 2-mercaptoethanol, 0.004% bromophenol blue, and 0.125 mol/L Tris-HCl pH 6.8) and boiled at 95 °C for 5 min. The prepared samples were loaded onto gels consisting of a 5% stacking gel and a 12% resolving gel. Electrophoresis was performed using a Mini-PROTEAN Tetra Cell electrophoresis system (Bio-Rad Laboratories, Hercules, CA, USA). Electrophoresis was initially performed at a constant voltage of 80 V for 30 min. Once the bromophenol blue dye front entered the resolving gel, the voltage was increased to 120 V, and electrophoresis was continued until the dye front reached the bottom edge of the gel. After electrophoresis, the gels were stained with Coomassie Brilliant Blue R-250 solution and destained with destaining solution until clear protein bands were obtained. Finally, the gels were scanned using a gel imaging system (Bio-Rad Laboratories, Hercules, CA, USA) to analyze changes in the molecular weight distribution of the protein bands [6].

### 2.7. Fourier Transform Infrared (FTIR) Spectroscopy

Fourier transform infrared spectroscopy (FTIR) was employed to analyze changes in the secondary structure of MPs. Freeze-dried MPs powder (2 mg) was thoroughly mixed and ground with 200 mg of dried KBr powder, and the mixture was compressed into transparent pellets. Spectral scanning was performed at room temperature over the wavenumber range of 400 to 4000 cm^−1^, with a resolution of 4 cm^−1^ and 64 accumulated scans. The amide I region (1600–1700 cm^−1^) was curve-fitted using PeakFit software (Version 4.0, SeaSolve Software Inc., Framingham, MA, USA), and the integrated component areas were used to estimate the relative contents of α-helix, β-sheet, β-turn, and random-coil structures [6,7].

### 2.8. Intrinsic Tryptophan Fluorescence Spectroscopy

Endogenous tryptophan fluorescence spectra were measured using a fluorescence spectrophotometer (F-7100 Hitachi, Inc., Tokyo, Japan) to characterize changes in the tertiary structure of MPs. The MPs solution was diluted to a concentration of 0.5 mg/mL, and the excitation wavelength was set at 280 nm. Emission spectra were recorded over the range of 300–400 nm, with the slit width set at 5 nm [6,7].

### 2.9. Determination of Intermolecular Forces

A selective solvent extraction method was used with slight modifications [6,12]. 3.0 g of each MPs sample was accurately weighed and mixed with 17 mL of one of the following extracting solutions: SA, 0.05 mol/L NaCl; SB, 0.6 mol/L NaCl; SC, 0.6 mol/L NaCl containing 1.5 mol/L urea; SD, 0.6 mol/L NaCl containing 8.0 mol/L urea; and SE, 0.6 mol/L NaCl containing 8.0 mol/L urea and 0.05 mol/L β-mercaptoethanol. The mixtures were homogenized at 10,000 rpm for 1 min in an ice bath and stirred continuously at 4 °C for 1 h. They were then centrifuged at 12,000× *g* for 15 min at 4 °C, and the protein concentration in the supernatant was determined using the BCA assay. The relative contributions of different intermolecular forces were calculated based on the differences in soluble protein contents among the extracting solutions as follows: Ionic bonds = SB − SA, Hydrogen bonds = SC − SB, Hydrophobic interactions = SD − SC, and Disulfide bonds = SE − SD.

### 2.10. Determination of Protein Oxidation

The extent of malondialdehyde-induced protein oxidation was evaluated by determining the contents of reactive carbonyl groups (CB) and total sulfhydryl (SH) groups. The levels of CB and SH in the MPs system were quantified using commercial assay kits (Jiancheng Bioengineering Institute, Nanjing, China), strictly following the protocols provided by the manufacturer [7].

### 2.11. Determination of Hydrolyzed Amino Acids

Hydrolyzed amino acids were determined by acid hydrolysis followed by amino acid analyzer detection. Approximately 0.5 g of homogenized MPs sample was accurately weighed into a hydrolysis tube, mixed with 25 mL of 6 mol/L HCl, degassed under vacuum for 30 min, flushed with nitrogen, and immediately sealed. The samples were hydrolyzed at 110 °C for 22 h and then diluted to 50 mL with ultrapure water. Subsequently, 2 mL of hydrolysate was evaporated to dryness at 60 °C under vacuum or nitrogen flow. The residue was reconstituted in 2 mL of 0.02 mol/L HCl and diluted tenfold. After filtration through a 0.22 μm aqueous membrane, the amino acid composition was analyzed using an amino acid analyzer (LA8080, Hitachi, Japan) [13].

### 2.12. High-Performance Liquid Chromatography (HPLC)

MPs were dissolved in 0.1 M sodium phosphate buffer containing 0.05% NaN_3_ at pH 6.9 to obtain a final protein concentration of 1 mg/mL. The solution was centrifugally filtered through a 0.22 μm Millipore membrane at 2800× *g*. Subsequently, 20 μL of the filtered sample was injected into a TSK G3000 SWXL size exclusion chromatography column (Tosoh Corporation, Tokyo, Japan) and eluted with degassed and filtered 0.1 M sodium phosphate buffer at pH 6.6, with a flow rate of 1 mL/min. Absorbance was monitored at 280 nm using a Gilson ultraviolet detector, and the detection time was set to 15 min [14].

### 2.13. Proton Nuclear Magnetic Resonance (^1^H-NMR) Analysis

The treated MPs samples were lyophilized, and 10 mg of each freeze-dried sample was accurately weighed and dissolved in 550 μL of D_2_O. The mixture was thoroughly vortexed to obtain a homogeneous sample and then transferred into a 5 mm NMR tube. One-dimensional ^1^H-NMR spectra were acquired using an Avance III 400 MHz NMR spectrometer (Bruker BioSpin, Rheinstetten, Germany) equipped with a 5 mm QXI room-temperature probe. Water signal suppression was achieved using a presaturation pulse sequence. The acquisition parameters were set as follows: 128 scans, 4 dummy scans, a relaxation delay of 5 s, a presaturation frequency centered at 4.78 ppm, a time domain of 32 K, a spectral width of 20 ppm, and a flip angle of 45°. The free induction decays were Fourier-transformed after multiplication by an exponential window function with a line-broadening factor of 0.3 Hz. Changes in characteristic proton signals were subsequently analyzed to characterize MDA-induced alterations in the proton microenvironment and structure of MPs [15,16].

### 2.14. Redox Proteomic Analysis

#### 2.14.1. Protein Extraction and Biotin Labeling

For redox proteomic analysis, the control group (0 mM MDA) and the 10 mM MDA-treated group were selected, with three independent biological replicates per group. MPs samples were lysed in SDT buffer, sonicated on ice, and centrifuged at 14,000× *g* for 15 min at 4 °C. Protein concentration was determined by the BCA assay. Free thiols were blocked with 20 mM NEM at 60 °C for 30 min, and residual NEM was removed by TCA-acetone precipitation. The precipitated proteins were reduced with 5 mM TCEP in PBS containing 4% SDS at 50 °C for 1 h, followed by repeated precipitation. The reduced thiol sites were labeled with Biotin-HPDP at 25 °C for 3 h. Labeled proteins were concentrated, acetone-precipitated overnight in darkness, digested with trypsin for 12 h, desalted on C_18_ cartridges, and lyophilized.

#### 2.14.2. Enrichment of Oxidized Peptides

Biotin-labeled peptides were dissolved in loading buffer and incubated with streptavidin agarose resin for 3 h at room temperature. After washing with PBS, captured peptides were eluted twice with 50 mM NH_4_HCO_3_ containing 5 mM TCEP. The pooled eluates were alkylated with 20 mM IAA in the dark for 1 h, desalted, and freeze-dried.

#### 2.14.3. LC-MS/MS Analysis

Peptides were separated using an Easy-nLC 1200 system on a C18 column at 300 nL/min with a 120 min acetonitrile gradient. MS/MS analysis was performed in positive-ion DDA mode over 350–1800 *m*/*z*. Full MS scans were acquired at 60,000 resolution, and the top 20 precursor ions were fragmented by HCD at 15,000 resolution, with an isolation window of 1.6 *m*/*z* and normalized collision energy of 28.

#### 2.14.4. Database Searching

Raw spectra were analyzed using Proteome Discoverer 2.4 against the UniProt Bos taurus database. A target-decoy strategy was applied, with the FDR threshold set at 1%. Protein quantification was based on razor and unique peptides.

### 2.15. Molecular Docking

The three-dimensional structure of MDA was obtained from the PubChem database (CID: 10964) and energy-minimized using Chem3D. The crystal structures of representative MPs components were retrieved from the UniProt (https://www.uniprot.org/, accessed on 24 August 2026) and RCSB Protein Data Bank databases (https://www.rcsb.org/, accessed on 24 August 2026). Prior to docking, water molecules and non-essential ligands were removed from the protein structures, followed by the addition of polar hydrogen atoms and charge assignment using AutoDock Tools (version 1.5.6, The Scripps Research Institute, La Jolla, CA, USA). Both receptor proteins and MDA were converted into PDBQT format for subsequent docking analysis. Docking simulations were conducted using AutoDock Vina (version 1.1.2, The Scripps Research Institute, La Jolla, CA, USA), with a grid box of 15 × 15 × 15 Å centered on the predicted active or oxidation-related binding regions. Semi-flexible docking was applied, while the remaining parameters were retained at their default settings. The docking complexes were converted from PDBQT to PDB format using Open Babel and visualized using PyMOL (version 2.5.0, Schrödinger, LLC, New York, NY, USA) to analyze binding energies.

### 2.16. Statistical Analysis

All measurements were carried out in triplicate from independent experiments, and data are presented as mean ± standard deviation (SD). Statistical analyses were performed using IBM SPSS Statistics software (version 26.0, IBM Corp., Armonk, NY, USA). Differences among treatments were assessed by one-way analysis of variance (ANOVA), followed by Tukey’s honestly significant difference (HSD) test. Statistical significance was defined at *p* < 0.05. Densitometric analysis of SDS-PAGE bands was conducted using ImageJ software (version 1.53t, National Institutes of Health, Bethesda, MD, USA). Graphs were generated using OriginPro 2021 software (OriginLab Corp., Northampton, MA, USA).

## 3. Results

### 3.1. MDA-Induced WHC and Changes in Water Distribution of MPs

**Figure 1:**
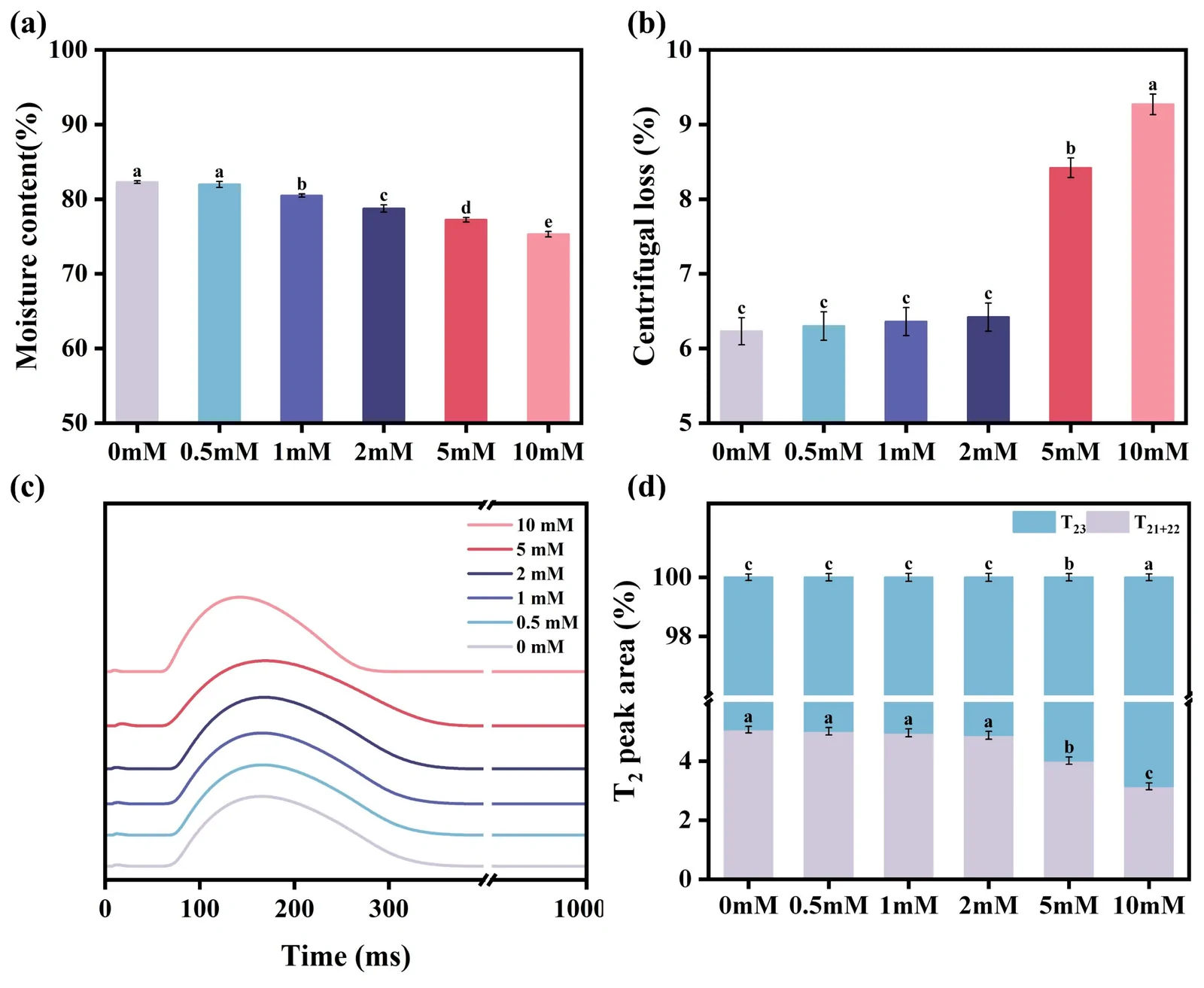
Effects of malondialdehyde on the WHC and water distribution of MPs. (**a**) Moisture content; (**b**) centrifugal loss; (**c**) LF-NMR transverse relaxation (T_2_) spectra; and (**d**) relative peak-area proportions of the combined bound- and immobilized-water fraction (T_21+22_) and free-water fraction (T_23_). Different lowercase letters indicate significant differences among MDA treatments (*p* < 0.05).

The effects of MDA treatment on the water-holding capacity (WHC) and water distribution of bovine MPs are shown in Figure 1. The moisture content remained unchanged after treatment with 0.5 mM MDA compared with that of the untreated control (*p* > 0.05). However, a significant decrease was observed when the MDA concentration reached 1 mM, and the moisture content subsequently declined in a concentration-dependent manner (*p* < 0.05). The moisture content decreased from 82.15% in the control to 75.12% at 10 mM MDA, corresponding to a reduction of 7.03 percentage points, or 8.56% relative to the control (Figure 1a). In contrast, the centrifugal loss showed no significant differences among the 0, 0.5, 1, and 2 mM MDA groups, with values ranging from 6.23% to 6.42% (*p* > 0.05; Figure 1b). When the MDA concentration increased to 5 mM, the centrifugal loss increased markedly to 8.42%, and a further increase to 9.27% was observed at 10 mM MDA (*p* < 0.05). The centrifugal loss in the 10 mM MDA group was 48.80% higher than that in the control. These results indicate that low MDA concentrations exerted limited effects on MPs’ water retention, whereas higher concentrations markedly increased water loss.

The LF-NMR T_2_ relaxation spectra were dominated by a broad long-relaxation component distributed mainly at approximately 80–300 ms (Figure 1c). Quantitative analysis of the relative peak areas further demonstrated MDA-induced redistribution of water types (Figure 1d). The relative proportion of free water, T_23_, showed no significant differences among the 0–2 mM MDA groups, increasing only slightly from 94.933% in the control to 95.127% at 2 mM MDA (*p* > 0.05). However, T_23_ increased significantly to 95.983% at 5 mM MDA and reached 96.853% at 10 mM MDA (*p* < 0.05). The 10 mM treatment therefore increased T_23_ by 1.920 percentage points relative to the control. Conversely, T_21+22_ exhibited an opposite trend to T_23_, decreasing significantly at 5 and 10 mM MDA to 4.017% and 3.147%, respectively (*p* < 0.05). Collectively, the decrease in moisture content, increase in centrifugal loss, and shift from the T_21+22_ fraction toward T_23_ demonstrate that high MDA concentrations increased water mobility and substantially reduced the WHC of bovine MPs.

### 3.2. Structural and Molecular Modifications of MPs Induced by MDA

**Figure 2:**
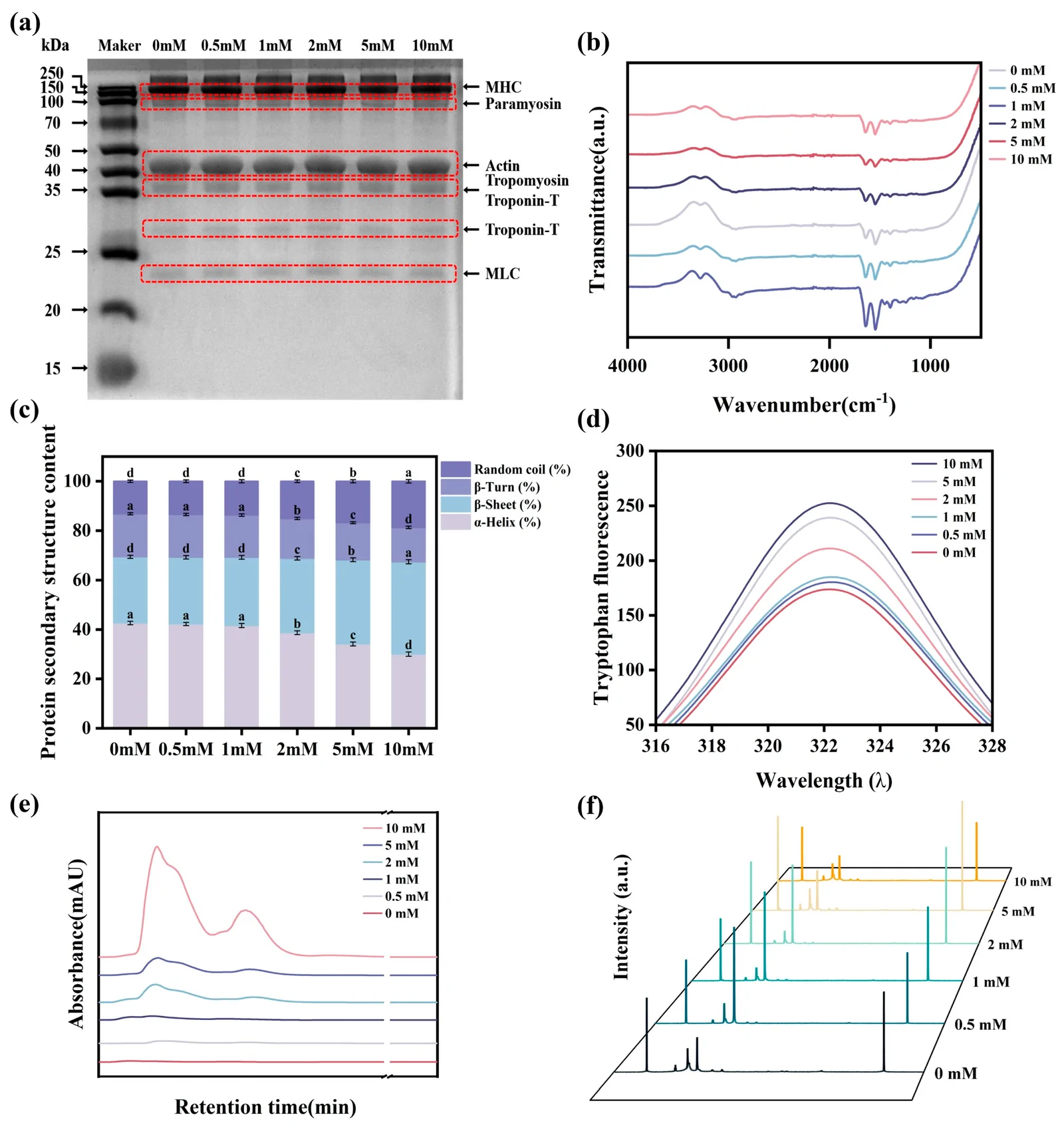
Effects of MDA on the structural and molecular characteristics of MPs. (**a**) SDS-PAGE profiles; (**b**) Fourier transform infrared spectra; (**c**) relative contents of α-helix, β-sheet, β-turn, and random coil structures; (**d**) intrinsic tryptophan fluorescence spectra; (**e**) high-performance liquid chromatography profiles; (**f**) stacked one-dimensional proton nuclear magnetic resonance (1H NMR) spectra. Different lowercase letters indicate significant differences among MDA treatments (*p* < 0.05). MHC, myosin heavy chain; MLC, myosin light chain.

The effects of MDA treatment on the structural and molecular characteristics of bovine MPs are shown in Figure 2. SDS-PAGE profiles remained generally similar across the 0–10 mM MDA treatments, with the major myosin heavy chain (MHC, 200 kDa) and actin (42 kDa) bands remaining clearly visible (Figure 2a). No pronounced accumulation of low-molecular-weight fragments was observed, indicating that extensive peptide-chain cleavage was not a dominant response under the present conditions. The FTIR spectra retained the characteristic absorption regions of MPs, including the broad amide A region at approximately 3200–3400 cm^−1^ and the amide I and amide II regions at approximately 1600–1700 and 1500–1600 cm^−1^, respectively (Figure 2b). Progressive changes in band shape and intensity were observed with increasing MDA concentration, particularly within the amide I region. Quantitative analysis further revealed a concentration-dependent rearrangement of MPs’ secondary structure (Figure 2c). The contents of α-helix, β-sheet, β-turn, and random coil did not differ significantly among the 0, 0.5, and 1 mM MDA groups (*p* > 0.05). At 2 mM MDA, α-helix content decreased from 42.60% to 38.70%, whereas β-sheet and random-coil contents increased from 26.80% and 13.20% to 30.10% and 15.10%, respectively (*p* < 0.05). At 10 mM MDA, α-helix and β-turn contents decreased to 30.00% and 13.90%, respectively, while β-sheet and random-coil contents increased to 37.40% and 18.70%. Compared with the control, the 10 mM treatment decreased α-helix content by 29.58% and increased β-sheet content by 39.55%, indicating a transition from an α-helix-rich structure toward β-sheet-rich and more disordered conformations. Intrinsic tryptophan fluorescence intensity increased progressively with increasing MDA concentration, particularly at 2–10 mM, whereas the maximum emission wavelength remained close to 322 nm (Figure 2d). The increase in fluorescence intensity without an evident shift in the emission maximum indicates that MDA altered the local microenvironment surrounding tryptophan residues, supporting the occurrence of tertiary-structure rearrangement.

HPLC was used to characterize MDA-induced changes in the chromatographic profiles of the washed MPs fractions (Figure 2e). The untreated control exhibited only a weak baseline response, whereas the chromatographic peak areas increased progressively with increasing MDA concentration. Only limited signals were observed in the 0.5 and 1 mM MDA groups, while more distinct peaks emerged at 2 mM MDA. The peak responses increased markedly in the 5 mM group and reached their highest levels in the 10 mM group, which displayed pronounced chromatographic signals in both the earlier- and later-eluting regions. These results demonstrate concentration-dependent alterations in the chromatographic characteristics of the washed MPs fractions following MDA treatment. The stacked one-dimensional ^1^H-NMR spectra further showed MDA-dependent changes in the proton spectral profiles of MPs (Figure 2f). The spectra of the 0.5 and 1 mM groups largely overlapped with that of the control, indicating that low concentrations of MDA produced only limited perturbations in the overall proton environment. More evident spectral changes emerged when the MDA concentration reached 2 mM, particularly within the aliphatic region at δ 0.50–2.20 ppm and the polar proton region at δ 2.20–4.50 ppm. These changes were characterized by variations in resonance intensity, progressive line broadening, and reduced spectral resolution. At higher MDA concentrations, especially 5 and 10 mM, the redistribution and broadening of resonance signals became more pronounced. Changes in the δ 0.50–1.80 ppm region indicate alterations in the microenvironment and mobility of hydrophobic aliphatic side chains, whereas variations in the δ 2.20–4.50 ppm region suggest perturbations involving polar side chains and the peptide backbone. Minor changes in the aromatic region at δ 6.50–8.50 ppm may further reflect altered exposure of aromatic residues during protein unfolding and aggregation. Collectively, these results demonstrate that low MDA concentrations caused limited structural changes, whereas MDA concentrations of 2–10 mM induced progressively greater secondary- and tertiary-structure rearrangement. The loss of α-helical structure, enrichment of β-sheet and random-coil conformations, and alterations in the local molecular environment indicate the formation of a less ordered and more heterogeneous MPs structure, consistent with the deterioration in water retention observed in Figure 1.

### 3.3. MDA-Induced Oxidation and Alteration of Intermolecular Forces in MPs

**Figure 3:**
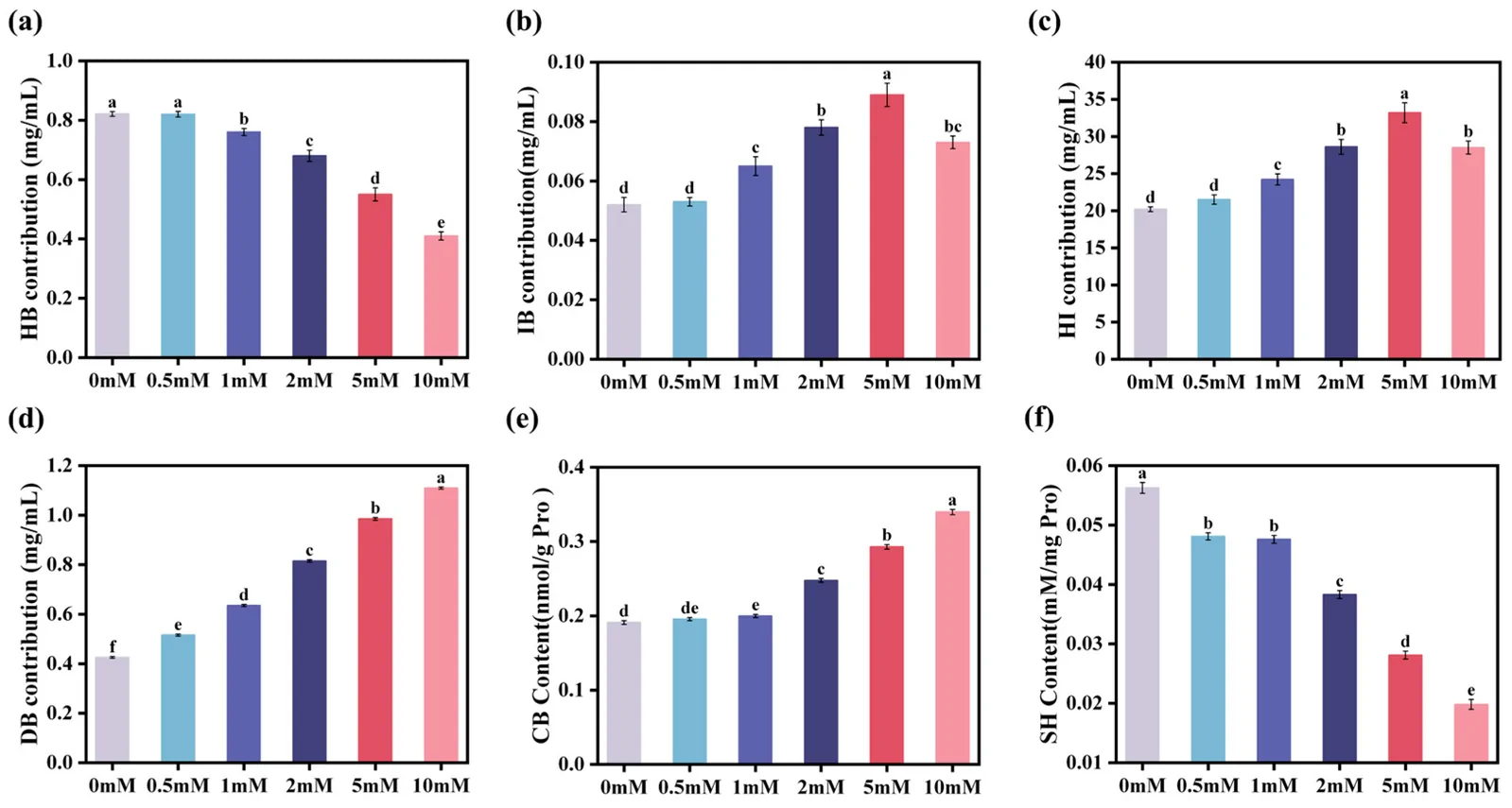
Effects of malondialdehyde on the intermolecular forces and oxidation indices of bovine myofibrillar proteins. (**a**) Hydrogen bonds; (**b**) Ionic bonds; (**c**) Hydrophobic interactions; (**d**) Disulfide bonds; (**e**) Carbonyl contribution; and (**f**) Total sulfhydryl contribution. Different lowercase letters indicate significant differences among MDA treatments (*p* < 0.05).

The effects of MDA treatment on intermolecular forces and protein oxidation of MPs are shown in Figure 3. The relative contribution of hydrogen bonds remained unchanged at 0.5 mM MDA (*p* > 0.05) but decreased progressively thereafter, from 0.821 mg/mL in the control to 0.410 mg/mL at 10 mM MDA (*p* < 0.05), corresponding to a 50.1% reduction (Figure 3a). In contrast, the relative contribution of ionic bonds increased with MDA concentration up to 5 mM, reaching a maximum of 0.089 mg/mL (*p* < 0.05), and subsequently decreased to 0.073 mg/mL at 10 mM (Figure 3b). Nevertheless, the value at 10 mM remained 40.4% higher than that of the control. Hydrophobic interactions exhibited a similar biphasic response, increasing from 20.20 mg/mL in the control to 33.20 mg/mL at 5 mM MDA and then decreasing to 28.50 mg/mL at 10 mM (Figure 3c, *p* < 0.05). By contrast, the relative contribution of disulfide bonds increased continuously with increasing MDA concentration, from 0.425 mg/mL in the control to 1.109 mg/mL at 10 mM MDA (*p* < 0.05), representing a 160.9% increase (Figure 3d).

Protein oxidation was further evaluated by measuring carbonyl and total sulfhydryl contents (Figure 3e,f). Carbonyl contribution increased progressively with increasing MDA concentration, particularly at concentrations of 2–10 mM. At 10 mM MDA, carbonyl contribution was 77.9% higher than that of the control (*p* < 0.05). Conversely, total sulfhydryl contribution decreased markedly after MDA treatment. No significant difference was observed between the 0.5 and 1 mM MDA groups (*p* > 0.05), whereas further increases in MDA concentration resulted in significant reductions in total sulfhydryl contribution. Compared with the control, treatment with 10 mM MDA decreased the total sulfhydryl contribution by 64.8%. Collectively, these results indicate that MDA treatment promoted protein oxidation and markedly altered the intermolecular interactions maintaining MPs’ structure, as evidenced by weakened hydrogen bonding, enhanced disulfide-associated interactions, and a transient increase in hydrophobic and ionic interactions.

### 3.4. MDA-Induced Changes in the Amino Acid Composition of MPs

The effects of MDA treatment on the amino acid composition of MPs are shown in Table 1. MDA caused residue-dependent changes in the amino acid profile, with the greatest decrease observed for Cys, followed by Met, His, Lys, and Tyr. Cys decreased markedly from 0.30 g/100 g in the control to 0.05 g/100 g at 10 mM MDA, corresponding to an 83.33% reduction, with the first significant decrease observed at 1 mM MDA (*p* < 0.05). Lys and His also decreased progressively across the MDA concentration range, from 2.02 to 1.58 g/100 g and from 0.68 to 0.53 g/100 g, respectively. Similarly, Met, Arg, and Tyr decreased from 0.99, 2.25, and 1.27 g/100 g in the control to 0.74, 2.08, and 1.02 g/100 g at 10 mM MDA, respectively. Relative to the control, the decreases in Lys, His, Met, Arg, and Tyr at 10 mM MDA were approximately 21.78%, 22.06%, 25.25%, 7.56%, and 19.69%, respectively. In comparison, Asp, Thr, Ser, Phe, and Pro showed relatively moderate decreases. Asp and Thr decreased significantly only at 10 mM MDA, whereas Ser decreased significantly at 5 and 10 mM MDA (*p* < 0.05). Glu remained unchanged across all MDA concentrations (*p* > 0.05), while Phe showed a significant reduction only at 10 mM MDA (*p* < 0.05). Gly, Ala, Val, Ile, and Leu remained unchanged throughout the treatments (*p* > 0.05). Collectively, these results demonstrate that the hydrolyzed amino acid profile of MPs responded to MDA in a residue-dependent manner, with Cys and several sulfur-containing, basic, and aromatic amino acids showing greater susceptibility to MDA treatment. These compositional changes are consistent with the progressive protein oxidation and sulfhydryl depletion observed in Figure 3.

### 3.5. Identification of MDA-Mediated Cysteine Oxidative Modifications in MPs

**Figure 4:**
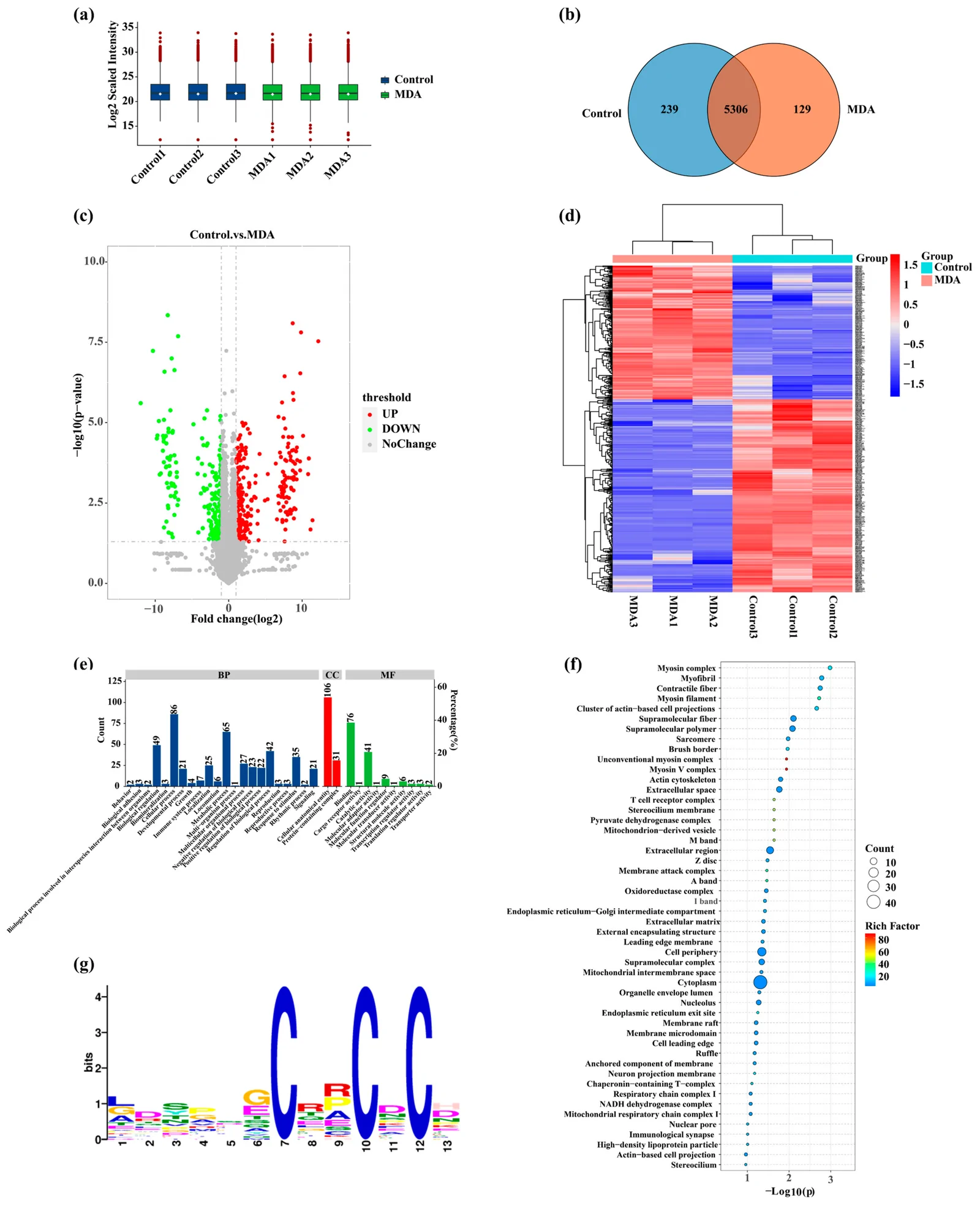
Quantitative proteomics analysis of cysteine oxidation modifications in proteins under MDA treatment. (**a**) Box plot of Log2-scaled intensity across different samples for data normalization and quality control. (**b**) Venn diagram showing the number of identified modified peptides. (**c**) Volcano plot of differentially oxidized peptides. In the volcano plot, red, green, and gray dots indicate upregulated, downregulated, and unchanged sites, respectively. (**d**) Hierarchical clustering heatmap of the significantly differentially oxidized peptides. In the heatmap, red and blue indicate relatively high and low normalized abundances, respectively. (**e**) Gene Ontology (GO) annotation classification of the differentially modified proteins. (**f**) Bubble chart showing the GO functional enrichment analysis of the differentially modified proteins. (**g**) Motif analysis of amino acid sequences surrounding the identified cysteine oxidation sites. BP: biological process, CC: cellular component, MF: molecular function. In the enrichment plot, bubble size represents the number of proteins assigned to each term, whereas bubble color represents the rich factor.

To comprehensively evaluate the effects of MDA treatment on oxidative modifications of protein cysteine residues, quantitative proteomic analysis was performed on the Control and MDA-treated groups. As shown in Figure 4a, the boxplots of log2-transformed intensity distributions exhibited a high degree of consistency across all samples, indicating good reproducibility of sample preparation and sufficient data quality for subsequent quantitative analysis. The Venn diagram (Figure 4b) showed that 5306 cysteine oxidation sites were commonly identified in both the Control and MDA groups, whereas 129 modified peptides were uniquely detected in the MDA group. Differential modification analysis was subsequently performed using a ≥2-fold change and *p* < 0.05 as the significance thresholds. As illustrated by the volcano plot (Figure 4c and Table S1), a total of 581 differentially abundant cysteine oxidation sites were identified, including 343 upregulated and 238 downregulated sites in the MDA group compared with the Control group. Furthermore, hierarchical clustering analysis (Figure 4d) revealed that the abundance profiles of the differentially modified peptides clearly separated the MDA-treated samples from the Control samples, demonstrating that MDA treatment induced a distinct and pronounced remodeling of protein cysteine oxidative modifications.

GO classification further indicated that the differential proteins were mainly involved in cellular processes, biological regulation, metabolic processes, responses to stimuli, and localization. At the molecular-function level, binding and catalytic activity represented the principal categories (Figure 4e). Gene Ontology cellular component enrichment analysis showed that the proteins containing differential cysteine sites were predominantly associated with the myosin complex, myofibril, contractile fiber, myosin filament, actin-based cell projections, supramolecular fibers and polymers, and the sarcomere (Figure 4f). Other enriched cellular components included the unconventional myosin complex, myosin V complex, actin cytoskeleton, A band, I band, Z disc, and M band. These enrichment patterns indicate that MDA-responsive cysteine sites were extensively distributed in proteins responsible for sarcomeric organization, contractile filament assembly, and cytoskeletal stability. To investigate the sequence preference of amino acids surrounding cysteine oxidation sites, motif enrichment analysis was performed on the identified modified peptides. Sequence motif analysis revealed a distinct amino acid enrichment pattern near the oxidation sites (Figure 4g). The most prominent feature was the strong conservation of cysteine (C) residues at positions 10 and 12 downstream of the centrally aligned cysteine residue (position 7), forming a characteristic C-x-x-C-x-C motif. In addition, weak enrichment of several other amino acids was observed at specific positions, including G and E at position 6 and R, P, and A at position 9; however, their degree of conservation was substantially lower than that of cysteine.

To further characterize cysteine redox changes in proteins closely associated with myofibrillar structure, 57 significantly altered cysteine sites corresponding to 34 UniProt entries were summarized in Table 2. All listed sites exhibited lower quantitative intensities in the MDA group than in the Control group, with Control-to-MDA fold changes ranging from 2.01 to 2714.74 (*p* < 0.05). These sites were distributed across major contractile and cytoskeletal proteins, including actin, myosin, α-actinin, filamin C, nebulin, myomesin, spectrin, connectin, smoothelin-like protein 2, and several LIM-domain-containing proteins. Several structural proteins contained multiple responsive cysteine sites. Four sites were identified in nebulin, including C420, C667, C1260, and C5040, while four sites were detected in myomesin 2 at C204, C678, C752, and C1396. Filamin C contained differential sites at C439, C1406, and C1654, and α-actinin-3 contained sites at C490, C494, and C593. Multiple cysteine sites were also detected in four-and-a-half LIM domains protein 1, spectrin β-chain, myosin XVIIIB, and other LIM-domain-containing proteins. The most pronounced decreases were observed at C251 of four-and-a-half LIM domains protein 3, C1406 of filamin C, C411 of smoothelin-like protein 2, C752 of myomesin 2, C21 of connectin, C1414 of myosin XVIIIB, and C2266 of spectrin α non-erythrocytic 1, with fold changes of 2714.74, 1119.07, 640.11, 526.23, 520.80, 451.84, and 422.48, respectively. In addition, marked decreases occurred at C258 of actin, gamma-enteric smooth muscle and C283 of creatine kinase M-type, with fold changes of 40.01 and 39.37, respectively. Collectively, these findings demonstrate that severe MDA treatment induced extensive site-specific remodeling of cysteine redox states in proteins maintaining myofibrillar architecture, which may contribute to the disruption of sarcomeric integrity and the deterioration of MPs WHC.

### 3.6. Molecular Docking of MDA near Redox-Proteomics-Identified Cysteine Sites

**Figure 5:**
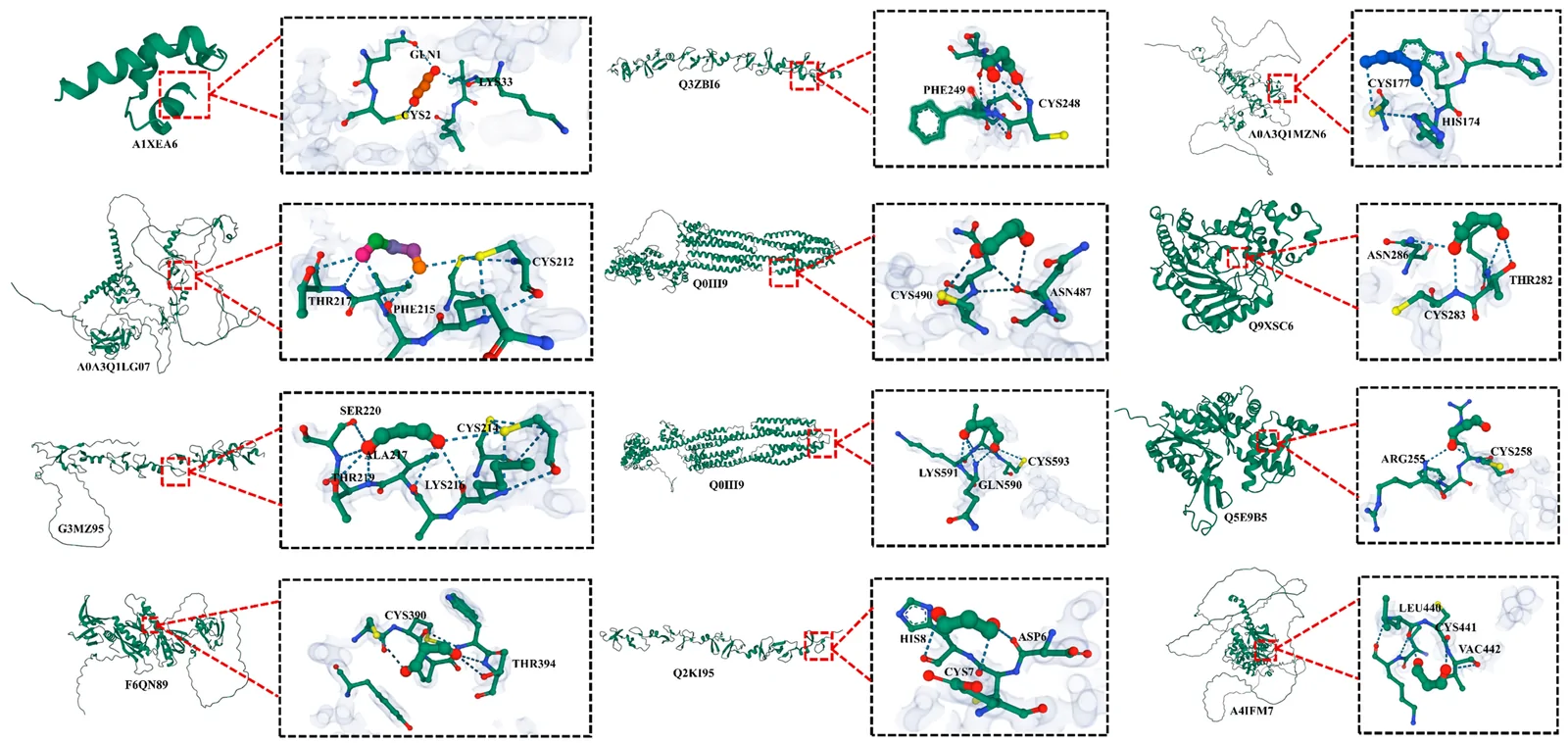
Molecular docking models illustrating the spatial conformations and binding interactions between MDA and specific Cys residues across 12 MP-related proteins. The comprehensive 3D protein structures are displayed alongside magnified, dashed-box views of the active docking centers and adjacent interacting amino acid residues, with dashed lines indicating key intermolecular interactions.

Redox proteomic analysis initially identified 57 MDA-responsive cysteine oxidation sites distributed among myofibrillar, sarcomeric, and cytoskeletal proteins (Table 2). These sites were mainly located in proteins involved in contractile filament organization and structural stabilization, including actin, α-actinin, myosin-associated proteins, LIM-domain-containing proteins, and other cytoskeletal components. Site-directed molecular docking was performed to evaluate the potential association of MDA with 12 representative cysteine-containing regions selected from the redox proteomic analysis (Figure 5 and Table 3). The predicted Vina scores ranged from −1.8 to −3.3 kcal/mol, indicating relatively weak but energetically permissible non-covalent interactions between MDA and the local microenvironments surrounding the selected cysteine residues. Among the evaluated sites, actin C258 showed the most favorable docking score of −3.3 kcal/mol. Alpha-actinin-3 C490 and C593 and actin-binding LIM protein family member 2 C177 each exhibited a Vina score of −2.2 kcal/mol, followed by four-and-a-half LIM domains protein 1 C214 and syntrophin β2 C390, both with scores of −2.1 kcal/mol. The remaining sites showed docking scores between −1.8 and −2.0 kcal/mol. MDA also exhibited predicted interactions with several cysteine-rich LIM-domain proteins. The three-dimensional molecular models (Figure 5) visually corroborate these quantitative findings, revealing that MDA could adopt predicted poses in close proximity to the selected cysteine-containing regions. The magnified docking poses illustrate the specific spatial orientations, showing that the interactions are largely stabilized by hydrogen bonding between MDA and the amino acid side chains adjacent to the targeted cysteines. Overall, the consistent localization of MDA close to the target Cys residues supports the spatial feasibility of MDA–thiol interactions and suggests that neighboring polar, basic, aromatic, and hydrophobic residues may assist ligand recognition and orientation.

## 4. Discussion

In postmortem beef, malondialdehyde should be regarded not merely as an indicator of lipid oxidation, but as a reactive intermediate generated within a self-amplifying lipid–heme–protein oxidation network. Following exsanguination, the cessation of oxygen and nutrient supply progressively weakens cellular redox homeostasis, whereas mitochondria, myoglobin, transition metals, and polyunsaturated membrane lipids remain capable of participating in oxidative reactions. Myoglobin autoxidation produces metmyoglobin and reactive hypervalent heme species, while subsequent disruption of the porphyrin structure releases heme or non-heme iron that catalyzes further radical formation. Polyunsaturated fatty acids are consequently converted into lipid radicals and hydroperoxides, whose decomposition produces secondary carbonyl compounds, including MDA [7,17,18]. MDA can, in turn, react with nucleophilic protein residues and enhance myoglobin- and iron-dependent reactive oxygen species generation, thereby connecting lipid peroxidation with myofibrillar protein oxidation [7,18]. This feedback process is relevant to water retention because water in muscle is predominantly confined within the myofibrillar lattice, and its mobility depends on filament charge, interfilament spacing, protein conformation, and the structural constraints imposed by cross-links. Oxidation can exert two opposing effects. Modification of ionizable residues may increase the net charge and osmotic swelling pressure of myofilaments. In contrast, covalent cross-linking and aggregation restrict filament expansion and reduce the space available for water immobilization [19,20]. The final effect on water-holding capacity is determined by which process becomes dominant rather than by oxidation intensity alone. In intact postmortem beef, haem-mediated protein oxidation has been directly associated with increased water mobility and reduced water-holding capacity, supporting MDA as an important molecular link between postmortem oxidative metabolism and water loss [7,19].

The water-related results indicate that MDA affects water properties only when its concentration exceeds a certain threshold, rather than causing a gradual linear change (Figure 1). Moisture retention began to decrease at 1 mM MDA, whereas centrifugal loss and the redistribution of relatively restricted water toward the T_23_ fraction became pronounced only at 5–10 mM. This finding suggests that changes in the protein’s water-binding sites may have preceded the pronounced loss of network-level water retention. At relatively low MDA concentrations, limited side-chain modification and partial protein unfolding may expose charged or polar groups. Oxidation-induced loss of positively charged residues may also increase the net negative charge of the filaments. These effects could maintain electrostatic repulsion and filament swelling, at least partly compensating for the early formation of protein cross-links [21,22]. Once MDA accumulated beyond a critical level, however, the inhibitory effects of aggregation and covalent restriction became predominant. The resulting protein assemblies would resist lateral expansion, reduce the accessible surface available for protein–water hydrogen bonding, and facilitate the transfer of immobilized water into a more mobile state [23]. Thus, the marked increase in centrifugal loss at 5–10 mM reflects not simply “more protein oxidation,” but a transition from locally modified yet mechanically functional MPs to a rigid and spatially constrained matrix unable to retain water under external force.

Cysteine redox remodeling provides a molecular link between MDA-induced chemical modification and the progressive loss of structural flexibility in MPs. This conclusion is supported by multiple lines of evidence from the present study. The apparent Cys content decreased from 0.30 to 0.05 g/100 g after 10 mM MDA treatment, while total sulfhydryl content decreased by 64.8% and the apparent contribution of disulfide bonds increased by 160.9% (Table 1 and Figure 3). These parallel changes indicate a substantial depletion of cysteine residues from their native thiol state. However, the decrease in sulfhydryl groups should not be attributed solely to disulfide formation. Cysteine thiolates are highly redox-sensitive and may undergo reversible oxidation to sulfenic acid and disulfides or further oxidation to sulfinic and sulfonic derivatives under severe oxidative conditions [24]. Moreover, because reduced thiols were first blocked with NEM and oxidized thiols were subsequently reduced with TCEP and labeled with Biotin-HPDP in the present redox-proteomic workflow, changes in site intensity principally reflect alterations in the TCEP-recoverable cysteine redox pool, rather than direct quantitative measurement of a single oxidation product. Therefore, the lower abundance of the 57 selected cysteine sites after MDA treatment should not simply be interpreted as “increased oxidation”; instead, it may indicate conversion of these residues into less reducible oxidation states, stable thiol modifications, or conformational states that affect peptide recovery or labeling. Importantly, these alterations were not randomly distributed. The differentially modified proteins were strongly enriched in the myofibril, myosin complex, contractile fiber, sarcomere, Z-disc, A-band, I-band, and M-band, while the 57 structurally relevant sites occurred in actin, myosin, α-actinin, nebulin, myomesin, filamin C, spectrin, and connectin (Table 2). Similar redox-proteomic studies have identified myosin, actin, nebulin, titin, and α-actinin as oxidation-sensitive components of MPs, indicating that cysteine redox changes preferentially affect proteins responsible for filament organization and mechanical continuity [6,25,26]. Particularly noteworthy was the enrichment of a C-x-x-C-x-C-type cysteine-rich sequence environment together with the occurrence of multiple responsive sites in FHL and other LIM-domain proteins (Figure 4). LIM domains are zinc-binding structural modules in which conserved Cys and His residues coordinate Zn^2+^; modification of these residues can therefore disturb domain folding and protein–protein interactions rather than merely generate additional disulfide bonds [27]. Thus, the biological significance of cysteine remodeling lies not only in the loss of thiols themselves, but in its localization to structural nodes that control filament connectivity, sarcomeric elasticity, and the physical constraints governing myofibrillar swelling.

These cysteine redox alterations occurred together with residue-dependent decreases in the hydrolyzed amino acid profile and collectively drove the transition of MPs from a hydration-compatible conformation toward a structurally constrained aggregated state. Lys decreased from 2.02 to 1.58 g/100 g (21.78%), while His and Arg decreased by 22.06% and 7.56%, respectively (Table 1). This residue selectivity is chemically meaningful because MDA readily reacts with protein amino groups, and proteomic analysis of MDA-treated beef MPs has directly identified Schiff-base and dihydropyridine-type modifications of Lys, with myosin—particularly its tail region—being a major modification target [8]. Consistently, the carbonyl content in the present study increased by 77.9%, indicating progressive incorporation and/or generation of carbonyl functionalities (Figure 3). These residue-level reactions provide a mechanistic explanation for the coordinated changes in intermolecular forces and protein conformation. Hydrogen-bond-associated interactions decreased from 0.821 to 0.410 mg/mL, while α-helix content declined from 42.60% to 30.00% and β-sheet content increased from 26.80% to 37.40% (Figure 2). Because α-helical coiled-coils are central structural elements of myosin and several sarcomeric proteins, disruption of their hydrogen-bonding network would reduce conformational stability and expose previously buried side chains. This interpretation is supported by the increase in intrinsic tryptophan fluorescence and by the initial enhancement of hydrophobic interactions, which reached their maximum at 5 mM MDA. Once oxidation became more extensive, however, hydrophobic interactions declined despite further increases in carbonyl formation and disulfide-associated interactions. Rather than indicating structural recovery, this biphasic behavior likely reflects a transition from unfolding to aggregation: moderate modification exposes hydrophobic regions, whereas advanced cross-linking subsequently buries or immobilizes these residues within compact protein assemblies. Similar sequential unfolding-aggregation behavior has been observed in MDA-modified MPs and myosin [28,29,30]. The persistence of MHC and actin bands in SDS-PAGE indicates that extensive backbone fragmentation was not the principal pathway in the present system; instead, the combined increase in disulfide-associated interactions and MDA-sensitive amino acid modification favors conformational rearrangement and cross-linking. Moreover, the chromatographic changes that remained after three washing cycles support persistent alteration of the MPs fraction, although the size-exclusion signal detected at 280 nm cannot by itself distinguish MDA adduction from changes in protein aggregation state (Figure 2). Taken together, the chemical data suggest that cysteine redox remodeling and MDA-mediated amino-group modification operated concurrently, progressively replacing the native hydrogen-bond-stabilized and dynamically flexible structure with a β-sheet-enriched, aggregated architecture in which fewer polar groups and less protein surface remained accessible for hydration.

The convergence of redox proteomics, molecular docking, structural measurements, and water-distribution analysis further suggests that MDA-induced deterioration of WHC develops through a concentration-dependent transition from molecular modification to mechanical restriction of the myofibrillar matrix. Among the 12 redox-responsive cysteine regions examined by docking, actin C258 showed both a pronounced decrease in redox-proteomic signal (Control/MDA fold change = 40.01) and the most favorable MDA docking score (−3.3 kcal/mol). MDA was also positioned close to α-actinin-3 C490 and C593 and actin-binding LIM protein family member 2 C177, each with a predicted score of −2.2 kcal/mol (Table 2 and Table 3). Although these binding energies represent relatively weak non-covalent association and cannot demonstrate formation of MDA-Cys covalent adducts, the agreement between proteomic responsiveness and structural accessibility indicates that these regions constitute plausible pre-reaction recognition environments for MDA. Previous molecular studies likewise showed that MDA can be stabilized near myosin residues through hydrogen-bonding and hydrophobic interactions before subsequent oxidative or covalent modification [20,31]. Actin and α-actinin are particularly relevant to WHC because they participate directly in thin-filament and Z-disc organization; modification of such structural proteins, together with nebulin, myomesin, filamin, spectrin, and connectin, would progressively increase the constraints opposing lateral filament movement. This provides a mechanistic explanation for the apparent threshold observed in water retention. At 0.5–1 mM MDA, susceptible amino acids were already being depleted, but the secondary structure and centrifugal loss remained largely unchanged, indicating that the myofibrillar matrix could initially accommodate limited chemical modification. At approximately 2 mM, α-helical loss, β-sheet enrichment, altered fluorescence, and changes in intermolecular forces became evident, marking the onset of higher-order conformational destabilization. Only when MDA reached 5–10 mM did structural restriction become sufficiently extensive to produce a pronounced functional consequence: centrifugal loss increased from approximately 6.23% to 9.27%, whereas the combined T_21+22_ fraction decreased from 5.067% to 3.147% and T_23_ increased from 94.933% to 96.853% (Figure 1). This delayed water response is consistent with the concept that WHC is determined by competition between factors that favor myofibrillar swelling, such as changes in filament charge, and structural constraints generated by cross-linking and aggregation [20]. At low oxidation intensity, these processes may partially compensate for one another, whereas at high MDA concentrations, cysteine-centered redox remodeling, disulfide-associated restriction, carbonyl–amine reactions, and hydrophobic aggregation collectively overwhelm the ability of the filament lattice to expand. Consequently, water originally constrained within the MPs matrix becomes increasingly mobile and susceptible to centrifugal removal. The present results therefore support a hierarchical mechanism in which site-specific chemical modification precedes conformational destabilization, conformational destabilization promotes intermolecular association, and only after aggregation reaches a critical level does the loss of myofibrillar flexibility translate into substantial water migration and deterioration of WHC.

The present study verifies, using an isolated bovine myofibrillar protein model, that MDA is an important factor contributing to the deterioration of water-holding capacity and provides a mechanistic basis for developing more targeted strategies to preserve water retention in meat. One practical direction is to limit MDA formation and accumulation by suppressing lipid peroxidation during postmortem storage and processing. Improved temperature control, reduced exposure to oxygen and light, regulation of heme- and transition-metal-mediated oxidation, and the appropriate application of antioxidant systems may help interrupt the lipid oxidation–protein oxidation cascade before extensive structural damage to MPs occurs [32,33]. Another potential strategy is to reduce the reactivity of accumulated carbonyl compounds toward protein nucleophiles. Carbonyl-scavenging compounds or competitive nucleophiles may intercept reactive aldehydes before they modify critical amino acid residues; for example, L-lysine has been reported to alleviate MDA-induced oxidative damage and aggregation of MPs and improve their functional properties [34]. Preservation of cysteine redox homeostasis may also be important, because excessive thiol oxidation and disruption of cysteine-rich structural regions can alter protein–protein interactions and restrict the conformational flexibility required for myofibrillar swelling. Antioxidant interventions designed to protect redox-sensitive cysteine residues and maintain an appropriate thiol-disulfide balance may thus complement conventional approaches aimed at reducing overall oxidative stress. Maintaining WHC may additionally depend on an appropriate balance between structural stability and myofibrillar extensibility, since excessive cross-linking restricts filament expansion, whereas controlled postmortem proteolysis of cytoskeletal and Z-disc proteins can partially release these constraints and facilitate water retention [35]. Collectively, improvement of meat WHC may be achieved through coordinated control of lipid oxidation, reactive carbonyls, cysteine redox status, and myofibrillar structural remodeling, while the MDA-responsive cysteine sites identified here may serve as useful molecular targets or biomarkers for evaluating the effectiveness of such interventions.

## 5. Conclusions

This study shows that MDA reduces the WHC of bovine MPs in a concentration-dependent manner. Pronounced water migration and centrifugal loss at 5–10 mM MDA coincided with α-helix loss, β-sheet enrichment, weakened hydrogen-bond-associated interactions, and increased disulfide-associated restriction. Redox proteomics identified MDA-responsive cysteine sites in myofibrillar and cytoskeletal proteins, and docking indicated that MDA can associate near selected cysteine-containing regions, including actin C258. These findings link cysteine redox remodeling and protein aggregation with impaired water retention, while validation in whole-meat systems remains necessary.

## Figures and Tables

**Table 1:** Effects of MDA treatment on the hydrolyzed amino acid (g/100 g) composition of MPs.

Amino Acid	Full Name	0 mM	0.5 mM	1 mM	2 mM	5 mM	10 mM
Asp	Aspartic acid	0.52 ± 0.01 ^a^	0.52 ± 0.02 ^a^	0.51 ± 0.02 ^ab^	0.51 ± 0.02 ^ab^	0.50 ± 0.01 ^ab^	0.49 ± 0.02 ^b^
Thr	Threonine	1.78 ± 0.02 ^a^	1.78 ± 0.03 ^a^	1.77 ± 0.02 ^ab^	1.76 ± 0.03 ^ab^	1.74 ± 0.03 ^ab^	1.72 ± 0.03 ^b^
Ser	Serine	2.21 ± 0.02 ^a^	2.21 ± 0.01 ^a^	2.20 ± 0.03 ^ab^	2.18 ± 0.02 ^abc^	2.15 ± 0.03 ^bc^	2.13 ± 0.02 ^c^
Glu	Glutamic acid	4.00 ± 0.05 ^a^	4.02 ± 0.07 ^a^	3.99 ± 0.06 ^a^	3.97 ± 0.05 ^a^	3.94 ± 0.08 ^a^	3.91 ± 0.06 ^a^
Gly	Glycine	3.33 ± 0.03 ^a^	3.34 ± 0.04 ^a^	3.32 ± 0.02 ^a^	3.35 ± 0.03 ^a^	3.31 ± 0.04 ^a^	3.33 ± 0.03 ^a^
Ala	Alanine	11.79 ± 0.12 ^a^	11.82 ± 0.15 ^a^	11.76 ± 0.11 ^a^	11.81 ± 0.14 ^a^	11.74 ± 0.13 ^a^	11.77 ± 0.16 ^a^
Cys	Cysteine	0.30 ± 0.01 ^a^	0.28 ± 0.02 ^a^	0.20 ± 0.01 ^b^	0.16 ± 0.02 ^c^	0.09 ± 0.01 ^d^	0.05 ± 0.01 ^e^
Val	Valine	1.83 ± 0.02 ^a^	1.84 ± 0.03 ^a^	1.82 ± 0.01 ^a^	1.85 ± 0.02 ^a^	1.81 ± 0.03 ^a^	1.82 ± 0.02 ^a^
Met	Methionine	0.99 ± 0.02 ^a^	0.98 ± 0.03 ^a^	0.95 ± 0.02 ^ab^	0.90 ± 0.01 ^b^	0.82 ± 0.03 ^c^	0.74 ± 0.02 ^d^
Ile	Isoleucine	1.18 ± 0.02 ^a^	1.19 ± 0.01 ^a^	1.17 ± 0.03 ^a^	1.18 ± 0.02 ^a^	1.16 ± 0.02 ^a^	1.17 ± 0.01 ^a^
Leu	Leucine	2.22 ± 0.03 ^a^	2.23 ± 0.04 ^a^	2.21 ± 0.02 ^a^	2.24 ± 0.03 ^a^	2.20 ± 0.04 ^a^	2.21 ± 0.03 ^a^
Tyr	Tyrosine	1.27 ± 0.02 ^a^	1.26 ± 0.03 ^a^	1.23 ± 0.01 ^ab^	1.18 ± 0.02 ^b^	1.10 ± 0.03 ^c^	1.02 ± 0.02 ^d^
Phe	Phenylalanine	1.55 ± 0.02 ^a^	1.55 ± 0.01 ^a^	1.54 ± 0.03 ^ab^	1.53 ± 0.02 ^ab^	1.51 ± 0.02 ^ab^	1.49 ± 0.03 ^b^
Lys	Lysine	2.02 ± 0.03 ^a^	2.00 ± 0.04 ^a^	1.96 ± 0.02 ^ab^	1.88 ± 0.03 ^b^	1.74 ± 0.04 ^c^	1.58 ± 0.03 ^d^
His	Histidine	0.68 ± 0.02 ^a^	0.67 ± 0.01 ^a^	0.66 ± 0.03 ^a^	0.63 ± 0.02 ^ab^	0.58 ± 0.03 ^bc^	0.53 ± 0.02 ^c^
Arg	Arginine	2.25 ± 0.03 ^a^	2.24 ± 0.02 ^a^	2.22 ± 0.04 ^ab^	2.19 ± 0.03 ^ab^	2.14 ± 0.04 ^bc^	2.08 ± 0.03 ^c^
Pro	Proline	0.96 ± 0.02 ^a^	0.96 ± 0.03 ^a^	0.95 ± 0.01 ^a^	0.95 ± 0.02 ^a^	0.93 ± 0.03 ^a^	0.92 ± 0.02 ^a^

Note: Different lowercase letters within the same row indicate significant differences among treatments (*p* < 0.05).

**Table 2:** MDA-responsive cysteine oxidation sites in MPs identified by cysteine oxidation proteomics.

Num	Protein Position	Protein Name	Theo. MH+ [Da]	Sequence Length	Sequence Window	log_2_ Control	log_2_ MDA	FC	*p* Value
1	Q5E9B5-(C258)	Actin, gamma-enteric smooth muscle	3491.64324	30	VITIGNERFRCPETLFQPSFI	22.944 ± 0.221	14.564 ± 4.011	40.01	0.000
2	A0A3Q1LV98-(C439)	Filamin C	2667.32826	24	LEDKGDSTFRCTYRPVMEGPH	22.059 ± 0.298	17.801 ± 4.818	4.16	0.013
3	A0A3Q1M6W4-(C179)	Alpha-actinin-2	2779.2603	25	DERAIMTYVSCFYHAFAGAEQ	25.076 ± 0.058	22.141 ± 0.513	7.32	0.000
4	E1BF23-(C678)	Myomesin 2	3846.72752	32	EEDLLGYYVDCSVAGSNVWEP	20.965 ± 0.951	14.380 ± 3.691	16.71	0.045
5	F1MT60-(C667)	Nebulin	2968.39342	25	GSFEDPYQVHCLKISAQNSDK	23.767 ± 0.226	17.905 ± 4.899	12.37	0.001
6	A0A3Q1MAS7-(C411)	Smoothelin-like protein 2	2157.9692	18	AFTMAENLANCERLIEVEDMM	21.564 ± 0.177	12.249 ± 0.000	640.11	0.000
7	F1MT60-(C420)	Nebulin	2450.06522	20	GSYEDPYHTHCMRVSAQNSDK	26.626 ± 0.157	25.204 ± 0.106	2.69	0.001
8	E1BF23-(C1396)	Myomesin 2	2337.15838	20	IMEGKTLNLTCTVFGNPDPEV	24.175 ± 0.455	22.333 ± 0.116	3.69	0.013
9	Q9XSC6-(C283)	Creatine kinase M-type	2927.40796	26	WNEHLGYVLTCPSNLGTGLRG	24.950 ± 0.375	17.370 ± 4.551	39.37	0.002
10	Q0III9-(C593)	Alpha-actinin-3	2981.5037	26	LGIQGEIQKICQTYGLRPSST	28.137 ± 0.141	27.074 ± 0.172	2.09	0.001
11	Q0III9-(C490)	Alpha-actinin-3	2050.93209	17	YHEAASVNSRCQAICDQWDNL	24.770 ± 0.243	23.340 ± 0.047	2.72	0.003
12	Q0III9-(C494)	Alpha-actinin-3	2050.93209	17	ASVNSRCQAICDQWDNLGTLT	24.770 ± 0.243	23.340 ± 0.047	2.72	0.003
13	E1BF23-(C752)	Myomesin 2	3178.61762	29	SHPYGITLLNCDGHSMILGWK	21.277 ± 0.219	12.249 ± 0.000	526.23	0.000
14	A0A3Q1M1N1-(C670)	Uncharacterized protein	2509.24252	22	CLDLLSLSAACDALDQHNLKQ	20.055 ± 0.425	12.249 ± 0.000	229.95	0.003
15	F1MT60-(C5040)	Nebulin	2390.19348	19	DYRLHLHEWICHPDLQVNSHV	25.237 ± 0.278	14.660 ± 4.177	161.59	0.001
16	E1AXU0-(C737)	Cardiomyopathy associated protein 1	2229.04672	19	VHKFTWLFENCPMGSLAAESI	20.371 ± 0.132	12.249 ± 0.000	279.37	0.000
17	G3MZ95-(C138)	Four and a half LIM domains 1	1748.84181	14	TFVAKDNKILCNKCTTREDNP	21.394 ± 0.101	20.200 ± 0.566	2.18	0.006
18	G3MZ95-(C141)	Four and a half LIM domains 1	1748.84181	14	AKDNKILCNKCTTREDNPKCK	21.394 ± 0.101	20.200 ± 0.566	2.18	0.006
19	A0A3Q1LV98-(C1654)	Filamin C	1992.01536	19	VSIGGHGLGACLGPRIQIGEE	25.892 ± 0.167	24.643 ± 0.097	2.38	0.001
20	A4IFM7-(C441)	Myosin light chain kinase 2, skeletal/cardiac muscle	2513.39084	22	HLDLKPENILCVNTTGHLVKI	24.214 ± 0.321	22.336 ± 0.482	3.61	0.005
21	Q3ZBU0-(C104)	PDZ and LIM domain 5	2937.49861	27	PVQKPTVTSVCAETAQELAEG	26.104 ± 0.092	24.390 ± 0.035	3.29	0.000
22	E1BA80-(C1414)	Myosin XVIIIB	1637.79518	14	ADERFKGDVACQVLESERAER	21.052 ± 0.266	12.249 ± 0.000	451.84	0.001
23	Q9BE40-(C816)	Myosin-1	1584.79512	12	KMVERRESIFCIQYNVRAFMN	26.908 ± 0.221	25.277 ± 0.114	3.11	0.001
24	A6QPA6-(C814)	MYH3 protein	1598.81077	12	KMVQRRESIFCIQYNIRAFMN	26.581 ± 0.233	24.651 ± 0.130	3.83	0.001
25	A0A3Q1LV98-(C1406)	Filamin C	3631.71621	33	MSCKDNKDGSCTVEYIPFTPG	22.374 ± 0.117	12.249 ± 0.000	1119.07	0.000
26	E1BCU2-(C761)	Myomesin 3	3638.72202	30	VNQQPVPTQICKVSNLHEGHF	21.045 ± 0.626	14.751 ± 4.334	7.68	0.027
27	F1MT60-(C1260)	Nebulin	1507.61919	12	PDLPQFLQAKCNAYNLSDVCY	22.722 ± 0.334	21.010 ± 1.537	2.50	0.041
28	Q28086-(C21)	Connectin (Fragment)	2832.39129	25	SWGKPIYDGGCEIQGYIVEKC	21.265 ± 0.192	12.249 ± 0.000	520.80	0.000
29	A0A3Q1LG07-(C209)	Actin binding LIM protein 1	1350.59292	11	KDYQGLFGVKCEACHQFITGK	21.691 ± 0.046	20.454 ± 0.872	2.12	0.016
30	A0A3Q1LG07-(C212)	Actin binding LIM protein 1	1350.59292	11	QGLFGVKCEACHQFITGKVLE	21.691 ± 0.046	20.454 ± 0.872	2.12	0.016
31	A0A3Q1MXU7-(C1956)	Spectrin beta chain	1384.64131	12	IDARNDSFTTCIELGKSLLAR	20.790 ± 0.142	14.599 ± 4.072	8.32	0.003
32	E1BF23-(C204)	Myomesin 2	2070.05173	17	TVWERMSVKLCFTVQGFPTPV	22.774 ± 0.322	21.436 ± 0.170	2.56	0.012
33	Q17QE2-(C333)	LIM and cysteine-rich domains protein 1	1676.72678	14	DLAWHRKHFVCEGCEQQLGGR	25.823 ± 0.122	24.744 ± 0.053	2.12	0.000
34	Q17QE2-(C336)	LIM and cysteine-rich domains protein 1	1676.72678	14	WHRKHFVCEGCEQQLGGRAYI	25.823 ± 0.122	24.744 ± 0.053	2.12	0.000
35	A0A3Q1LXS3-(C158)	LIM and senescent cell antigen-like-containing domain protein	1766.84517	15	NNSWHPECFRCDLCQEVLADI	20.000 ± 0.324	14.336 ± 3.615	8.35	0.008
36	A0A3Q1LXS3-(C161)	LIM and senescent cell antigen-like-containing domain protein	1766.84517	15	WHPECFRCDLCQEVLADIGFV	20.000 ± 0.324	14.336 ± 3.615	8.35	0.008
37	Q3ZBI6-(C251)	Four and a half LIM domains protein 3	1552.6321	11	DRHWHHSCFSCARCSTSLVGQ	23.589 ± 0.525	12.249 ± 0.000	2714.74	0.011
38	F1MKE9-(C73)	Spectrin beta chain	1465.71173	12	WANSHLVHVSCRITDLYKDLR	22.966 ± 0.321	20.156 ± 1.667	4.49	0.015
39	A0A3Q1LWR2-(C803)	Uncharacterized protein	1480.72531	11	EQLNSRWIEFCQLLSERLNWL	20.972 ± 0.290	19.333 ± 0.088	3.15	0.003
40	E1BA80-(C1481)	Myosin XVIIIB	1559.66172	12	GADEWQMRFDCAQMENEFLRK	19.512 ± 0.063	12.249 ± 0.000	153.69	0.000
41	G3MZ95-(C211)	Four and a half LIM domains 1	1291.63981	10	TCHEAKFAKHCVKCNKAITSG	21.723 ± 0.477	20.295 ± 0.227	2.77	0.031
42	G3MZ95-(C214)	Four and a half LIM domains 1	1291.63981	10	EAKFAKHCVKCNKAITSGGIT	21.723 ± 0.477	20.295 ± 0.227	2.77	0.031
43	Q3ZC49-(C236)	Leucine-rich repeat-containing protein 39	1622.77642	14	TLWLQRNEITCLPETISSMKN	23.903 ± 0.270	22.152 ± 0.084	3.40	0.002
44	G3MZ95-(C114)	Four and a half LIM domains 1	1454.63823	10	HYKNRYWHDTCFRCSKCLQPL	25.355 ± 0.270	24.324 ± 0.430	2.01	0.021
45	A0A3Q1MXU7-(C170)	Spectrin beta chain	1277.63808	10	KSAKDALLLWCQMKTAGYPNV	22.227 ± 0.071	20.974 ± 0.093	2.38	0.000
46	F6QN89-(C390)	Syntrophin beta 2	1582.73925	12	VTEKDLLLYDCMPWTRDAWAS	19.365 ± 0.069	14.300 ± 3.553	5.69	0.008
47	Q0P585-(C52)	N-lysine methyltransferase SMYD2	1297.52009	10	VLTVSERGNHCEFCFARKEGL	22.303 ± 0.356	21.154 ± 0.194	2.25	0.018
48	Q0P585-(C55)	N-lysine methyltransferase SMYD2	1297.52009	10	VSERGNHCEFCFARKEGLSKC	22.303 ± 0.356	21.154 ± 0.194	2.25	0.018
49	A0A3Q1LWR2-(C1879)	Uncharacterized protein	892.40806	7	YKRQADDLLKCLDDIEKKLAS	20.963 ± 0.015	12.249 ± 0.000	420.03	0.000
50	A1XEA6-(C2)	Smooth muscle and non-muscle myosin alkali light chain peptide 6 (Fragment)	865.36548	7	CGDVMRALGQN	20.217 ± 0.269	14.759 ± 4.348	4.07	0.047
51	A0A3Q1LWN6-(C823)	Myosin heavy chain 11	839.408	7	LTAMKVIQRNCAAYLKLRNWQ	22.406 ± 0.137	18.263 ± 5.209	3.30	0.012
52	Q3ZBI6-(C248)	Four and a half LIM domains protein 3	1484.60588	11	SFEDRHWHHSCFSCARCSTSL	22.987 ± 0.271	21.025 ± 0.397	3.84	0.003
53	A0A3Q1MZN6-(C177)	Actin binding LIM protein family member 2	1084.51453	8	VALDKHWHLGCFKCKTCGKQL	23.076 ± 0.242	20.296 ± 0.249	6.87	0.001
54	Q2KI95-(C7)	Four and a half LIM domains protein 2	1679.66894	13	MTERFDCHHCEDSLFGR	22.020 ± 0.085	20.951 ± 0.062	2.10	0.000
55	Q2KI95-(C10)	Four and a half LIM domains protein 2	1679.66894	13	MTERFDCHHCEDSLFGRKYV	22.020 ± 0.085	20.951 ± 0.062	2.10	0.000
56	A0A3Q1N827-(C2266)	Spectrin alpha, non-erythrocytic 1	1999.88346	18	EVGDDLSGRSCMVEESGTLES	20.961 ± 0.215	12.249 ± 0.000	422.48	0.000
57	A6H7E3-(C259)	PDZ and LIM domain 1	779.34262	6	SIGNAQKLPMCDKCGTGIVGV	22.779 ± 0.059	21.701 ± 0.440	2.04	0.006

**Table 3:** Site-directed molecular docking of MDA at selected CYS oxidation sites in MPs.

Num	Protein Position	Protein Name	Sequence Window	Vina Score	Center (x, y, z)	Docking Size (x, y, z)
1	Q5E9B5-(C258)	Actin, gamma-enteric smooth muscle	VITIGNERFRCPETLFQPSFI	−3.3	−37, 2, 25	15, 15, 15
2	Q9XSC6-(C283)	Creatine kinase M-type	WNEHLGYVLTCPSNLGTGLRG	−1.8	−3, 3, −12	15, 15, 15
3	Q0III9-(C593)	Alpha-actinin-3	LGIQGEIQKICQTYGLRPSST	−2.2	−74, −12, 57	15, 15, 15
4	Q0III9-(C490)	Alpha-actinin-3	YHEAASVNSRCQAICDQWDNL	−2.2	−73, −12, 58	15, 15, 15
5	A4IFM7-(C441)	Myosin light chain kinase 2, skeletal/cardiac muscle	HLDLKPENILCVNTTGHLVKI	−2.0	11, 3, −17	15, 15, 15
6	A0A3Q1LG07-(C212)	Actin binding LIM protein 1	QGLFGVKCEACHQFITGKVLE	−1.8	−19, 7, −24	15, 15, 15
7	G3MZ95-(C214)	Four and a half LIM domains 1	EAKFAKHCVKCNKAITSGGIT	−2.1	−1, 3, 0	15, 15, 15
8	F6QN89-(C390)	Syntrophin beta 2	VTEKDLLLYDCMPWTRDAWAS	−2.1	−8, −3, −8	15, 15, 15
9	A1XEA6-(C2)	Smooth muscle and non-muscle myosin alkali light chain peptide 6 (Fragment)	CGDVMRALGQN	−2.0	1, −3, −1	15, 15, 15
10	Q3ZBI6-(C248)	Four and a half LIM domains protein 3	SFEDRHWHHSCFSCARCSTSL	−2.0	49, 6, −46	15, 15, 15
11	A0A3Q1MZN6-(C177)	Actin binding LIM protein family member 2	VALDKHWHLGCFKCKTCGKQL	−2.2	0, 4, −22	15, 15, 15
12	Q2KI95-(C7)	Four and a half LIM domains protein 2	MTERFDCHHCEDSLFGR	−1.9	36, 25, −66	15, 15, 15

## Data Availability

The original contributions presented in this study are included in the article/Supplementary Materials. Further inquiries can be directed to the corresponding authors.

## Supplementary Materials

The following supporting information can be downloaded at: https://www.mdpi.com/article/10.3390/foods15173022/s1, Table S1. Cysteine redox modification omics data.
